# Diagnostic Accuracy Performance of Fluorescence In Situ Hybridization (FISH) for Biliary Strictures: A Systematic Review and Meta-Analysis

**DOI:** 10.3390/jcm13216457

**Published:** 2024-10-28

**Authors:** Manik Aggarwal, Daniel M. Simadibrata, Benjamin R. Kipp, Larry J. Prokop, Emily G. Barr Fritcher, Amber Schneider, Matthew A. Cooley, Gregory J. Gores, John Eaton, Lewis R. Roberts, Vinay Chandrasekhara

**Affiliations:** 1 Division of Gastroenterology and Hepatology, Mayo Clinic, 200, First St SW, Rochester, MN 55905, USA; aggarwal.manik@mayo.edu (M.A.); simadibrata.daniel@mayo.edu (D.M.S.); gores.gregory@mayo.edu (G.J.G.); roberts.lewis@mayo.edu (L.R.R.); 2 Department of Laboratory Medicine and Pathology, Mayo Clinic, Rochester, MN 55905, USA; kipp.benjamin@mayo.edu (B.R.K.); barrfritcher.emily@mayo.edu (E.G.B.F.); schneider.amber@mayo.edu (A.S.); 3 Department of Library Services, Mayo Clinic, Rochester, MN 55905, USA; prokop.larry@mayo.edu; 4 Mayo Clinic Graduate School of Biomedical Sciences, Rochester, MN 55905, USA; cooley.matthew@mayo.edu

**Keywords:** fluorescence in situ hybridization, biliary stricture, diagnostic accuracy, meta-analysis

## Abstract

**Background and Aims**: This systematic review and meta-analysis aims to compare the performance of UroVysion^®^ FISH based on the different definitions of a positive result used in published literature with the goal of determining the optimal FISH definition for detecting pancreaticobiliary malignancy. **Methods**: A systematic literature search identified studies from database inception to Sept 2024 that evaluated the diagnostic performance of FISH in determining malignancy among patients with biliary strictures. All thresholds for positive FISH, as defined by the individual study, were included in this review. Subgroup analysis was performed based on the definitions of positive FISH as follows: (1) polysomy only; (2) polysomy, tetrasomy, or trisomy; and (3) polysomy or 9p deletion. **Results**: Eighteen studies comprising 2516 FISH specimens were analyzed, including 1133 (45.0%) with malignancy. Using a threshold for positivity as defined in individual studies, the overall sensitivity of FISH was 57.6% (95% confidence interval [CI], 49.4–65.4%), and the overall specificity was 87.8% (95% CI, 79.2–93.2%). Subgroup analysis showed that polysomy as the threshold for positive FISH yielded a sensitivity of 49.4% (95% CI, 43.2–55.5%), with an increased specificity of 96.2% (95% CI, 92.7–98.1%), while polysomy + tetrasomy/trisomy as positive FISH resulted in an increased sensitivity of 64.3% (95% CI 55.4–72.2%) but a decreased specificity of 78.9% (95% CI 64.4–88.5%). The addition of 9p deletion to polysomy as the criteria for a positive test resulted in a non-significant increase in sensitivity (54.7% (95% CI 42.4–66.5%) while maintaining specificity (95.1% (95% CI 84.0–98.6%). **Conclusions**: Based on these findings, polysomy only or polysomy/9p deletion should be considered as the criterion for defining a positive FISH test to improve diagnostic sensitivity while maintaining high specificity.

## 1. Introduction

Biliary stricture can arise from both benign (such as primary sclerosing cholangitis [PSC], chronic pancreatitis, infection, autoimmune cholangiopathy, and bile duct injury) and malignant conditions (such as cholangiocarcinoma [CCA] or pancreatic ductal adenocarcinoma [PDAC]) [1]. Most biliary strictures are considered malignant [2]; however, providing a definitive diagnosis of malignancy remains a clinical challenge. Several cytological and molecular techniques have been developed to aid in the diagnosis of malignant biliary strictures. These include traditional brush cytology, fluorescence in situ hybridization (FISH), and novel molecular techniques, such as methylated DNA markers and next-generation sequencing (NGS). Brushings of the biliary tract for cytology, a commonly performed diagnostic modality, is known to have poor sensitivity for detecting malignancy in biliary strictures [3]. FISH is a molecular cytogenetic technique that can be used to assess aneuploidy and has become a reliable diagnostic marker for detecting malignancy in patients with biliary strictures, with improved sensitivity over cytology while maintaining specificity [4,5]. Therefore, due to the increased sensitivity, current guidelines have suggested using multimodality analysis or tissue analysis over brush cytology alone [4,6].

Various FISH abnormalities are found in patients with biliary strictures diagnosed with malignancy, such as the presence of polysomy, tetrasomy, trisomy, and 9p deletion, among others [7]. Currently, there is a lack of consensus regarding the threshold or definition of positive FISH. These thresholds significantly affect the overall performance of FISH in evaluating biliary strictures. Thus, the lack of consensus has resulted in varying sensitivities and specificities of FISH across studies, potentially impacting patient management. This study aims to summarize the diagnostic performance of FISH for detecting malignancy in patients with biliary strictures. Furthermore, we aim to compare the performance of FISH, based on the different definitions used in published literature, with the goal of determining the optimal FISH definition for detecting pancreaticobiliary malignancy in patients undergoing endoscopic retrograde cholangiopancreatography (ERCP)-based tissue sampling.

## 2. Methods

This study was performed in accordance with the PRISMA guideline for diagnostic test accuracy (DTA) [8] and the Cochrane Handbook for Systematic Reviews of DTA [9]. The 27-item PRISMA diagnostic test accuracy checklist is provided in Supplementary Materials S1. Institutional review board approval was not needed for this study, due to the use of publicly available data.

### 2.1. Search Strategy and Selection Criteria

A comprehensive search of Ovid MEDLINE, Ovid Embase, Cochrane, and Scopus from database inception until September 2024 was performed by an expert librarian (JLP). (Search strategy is provided in Supplementary Materials S2) A priori inclusion criteria were full-text articles assessing the diagnostic accuracy of UroVysion^®^ FISH in adult (≥18 years of age) patients with biliary strictures who underwent ERCP-based brushing and comparing the findings with a clinical or histopathological diagnosis as the gold standard. Studies utilizing pancreaticobiliary (PB) FISH probe sets were excluded, due to the inherent differences between the FISH tests [10]. When studies had overlapping data, the study with the most complete and greater number of cases was included to avoid the double counting of subjects. This was evident in a few publications reported by the same institution [11,12]. Two studies were excluded, as they only included patients with inconclusive brush cytology, thus introducing selection bias [11,13]. There were no restrictions to the language of the study.

### 2.2. FISH Assay

FISH in each study was performed using a commercial kit (UroVysion^®^ probe set; Abbott Molecular Inc., Des Plaines, IL, USA) that contained labeled DNA probes in the pericentromeric regions of chromosomes 3, 7, and 17 and in the chromosomal band 9p21. Cells with a normal FISH pattern had 2 copies for each probe, which is consistent with a benign diagnosis. Three main abnormalities can be detected in cells using the FISH assay: (1) polysomy, 3 or more copies of 2 or more probes; (2) trisomy, 3 or more copies of 1 probe and 2 copies of the other 3 probes; and (3) tetrasomy, 4 copies of each probe. Additionally, the chromosomal band probe can show the loss of 9p21.

### 2.3. Study Outcomes

The outcome of interest was the diagnostic accuracy of FISH in evaluating biliary strictures, which included the sensitivity, specificity, positive likelihood ratio (PLR), negative likelihood ratio (NLR), diagnostic odds ratio (DOR), and area under the curve (AUC). The definition of a positive FISH result was determined per study definition, such as but not limited to (1) polysomy only, (2) polysomy/tetrasomy/trisomy, or (3) polysomy/9p deletion.

### 2.4. Study Selection, Data Extraction, and Quality Assessment

Two reviewers (M.A. and D.M.S) independently performed study adjudication, assessing the title, abstract, and full-text articles based on the eligibility criteria. For the included studies, data extraction was performed using Microsoft Excel (Version 2408 Build 16.0.17928.20114) to obtain key information on the study design and characteristics, patient demographics, definition of a positive FISH, diagnostic outcomes, and methodological quality. The risk of bias in the included studies was assessed using the QUADAS-2 tool recommended by the Cochrane Collaboration [14]. This tool assessed four domains: (1) methods of patient selection, (2) index test, (3) reference standard, and (4) flow of timing. Any discrepancies in the study selection, data extraction, and quality assessment processes were resolved by consensus between the two reviewers.

### 2.5. Statistical Analysis

A 2 × 2 contingency table was constructed using the extracted data to assess diagnostic performance. A DTA meta-analysis was performed using the “mada” package of the R program (version 0.5.11) (Vienna, Austria). The pooled sensitivity, specificity, PLR, NLR, and DOR with the 95% confidence interval (95% CI) were obtained using a bivariate random-effects model, as heterogeneity between studies was expected in the DTA meta-analysis. Univariate models or separate meta-analyses of sensitivity and specificity estimates are known to underestimate the test accuracy, due to the failure to account for the trade-off between sensitivity and specificity [9]. As endorsed by the Cochrane Handbook of Systematic Reviews of DTA studies [9], statistical heterogeneity was not assessed using the I^2^ statistic. I^2^ is often not appropriate for use in systematic reviews of DTA studies (particularly when the bivariate model has been used) because it is a univariate measure that does not account for threshold effects or the expected inverse correlation between sensitivity and specificity [15]. Tests used to measure heterogeneity are known to have low statistical power in DTA reviews [16]. The use of prediction ellipses around SROC curves (the approximate bivariate equivalent of a prediction interval) is a suitable alternative for graphically depicting heterogeneity in DTA meta-analyses. The magnitude of observed heterogeneity is best depicted graphically, where such relationships can be observed by the scatter of points and from the prediction ellipse. Consequently, the hierarchical summary receiver operating characteristic curve (SROC) with prediction ellipses were constructed, and the AUC value was determined. Subgroup analysis was performed based on the study definitions of positive FISH: (1) polysomy only, (2) polysomy/tetrasomy/trisomy, and (3) polysomy/9p deletion. Furthermore, we also analyzed the findings in patients with a known PSC diagnosis. Publication bias was assessed visually using a funnel plot and quantitatively analyzed using Deek’s linear regression test.

## 3. Results

### 3.1. Study Characteristics

**Figure 1:**
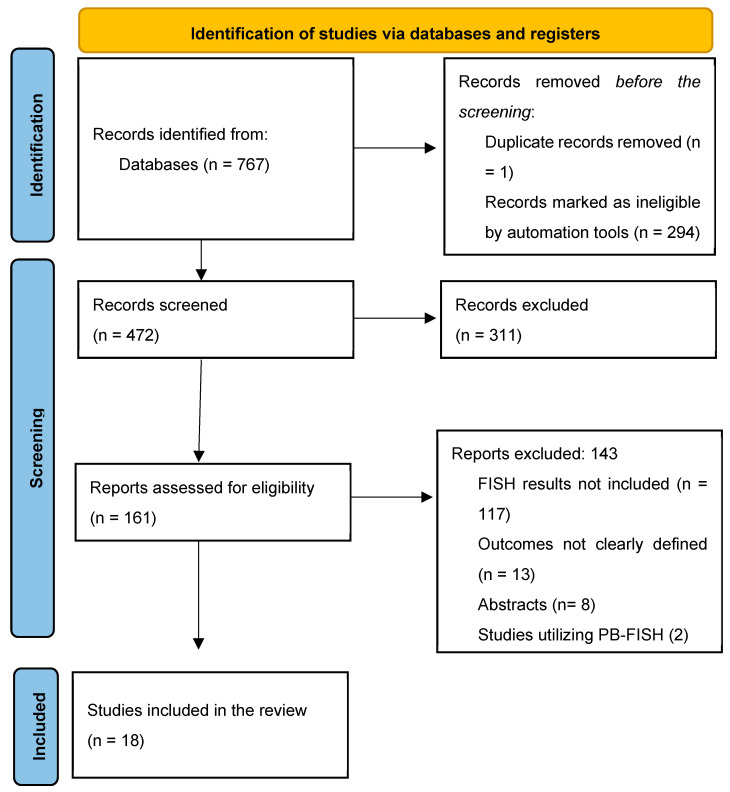
PRISMA flow chart for study selection.

**Figure 2:**
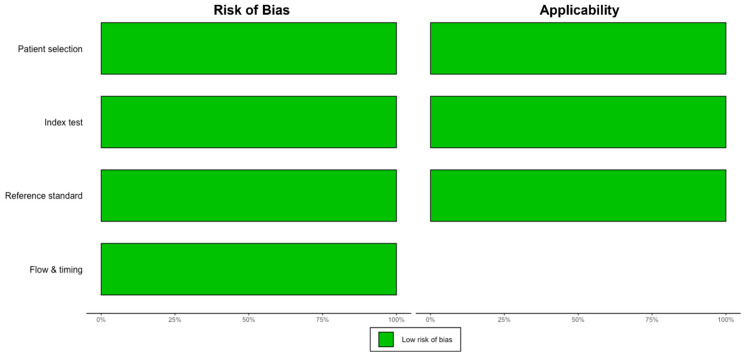
Summary of risk of bias based on QUADAS-2.

The initial search identified 472 titles, of which 157 were reviewed based on the full text for study eligibility (Figure 1). Overall, 18 studies (n = 2516 FISH specimens) were included, with malignancy identified in 1133 (45.0%) cases. All studies used the UroVysion^®^ (Abbott Molecular) probe set. Most of the studies were from the United States of America (n = 13) [5,6,17,18,19,20,21,22,23,24,25,26,27]; the remaining were from Poland [28], Italy [29], Thailand [30], China [31], and the Czech Republic [32]. All but two [22,30] were single-center studies. Only three studies had isolated data for PSC patients [5,18,22]. Studies used various thresholds/definitions for positive FISH, including but not limited to polysomy only (n = 13), polysomy/tetrasomy/trisomy (n = 8), and polysomy/9p deletion (n = 7). The overall risk of bias in the studies included was low (Figure 2, Supplementary Materials S3). Other study details and definitions of positive FISH are outlined in Table 1, Supplementary Materials S4.

### 3.2. Diagnostic Accuracy of FISH in Biliary Stricture

**Figure 3:**
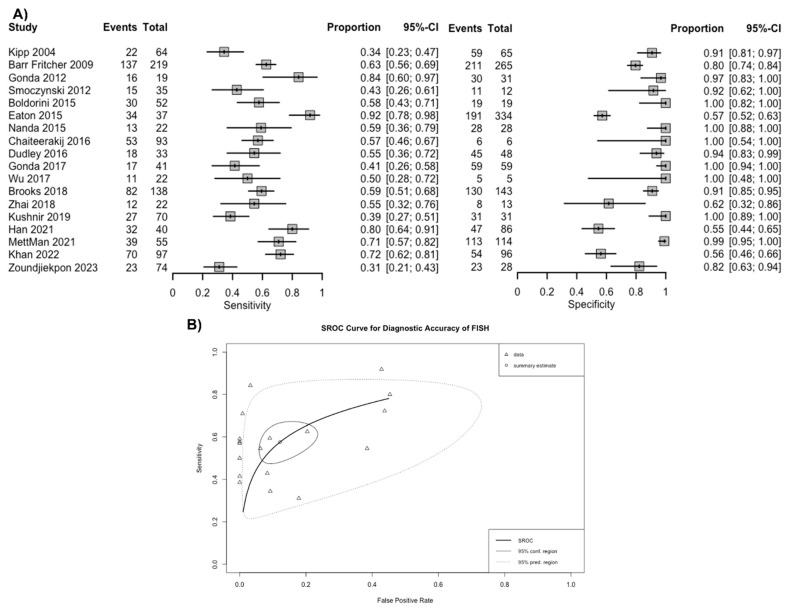
(**A**) Sensitivity and specificity of fluorescence in situ hybridization (FISH) for the diagnosis of malignancy in biliary strictures and (**B**) summary receiver operating curves. Footnote: The spread of individual data points (triangles) and the size of the 95% prediction region compared to the confidence region both suggest there is notable heterogeneity [5,6,17,18,19,20,21,22,23,25,26,27,28,29,30,31,32].

Overall, based on a positive threshold defined by individual studies, FISH had a sensitivity of 57.6% (95% CI 49.4–65.4%) and a specificity of 87.8% (95% CI 79.2–93.2%) for diagnosing malignancy in patients with biliary stricture (Figure 3A). The overall AUC was 0.76 (95% CI 0.74–0.78) (Figure 3B). The positive LR for FISH was 4.9 (95% CI 2.9–8.1), the negative LR was 0.49 (95% CI 0.40–0.57), and the DOR was 10.3 (95% CI 5.4–17.9). Based on the funnel plot and Deek’s linear regression test, publication bias was unlikely (*p* = 0.344) (Supplementary Materials S5).

### 3.3. Subgroup Analysis Based on FISH Definition

**Figure 4:**
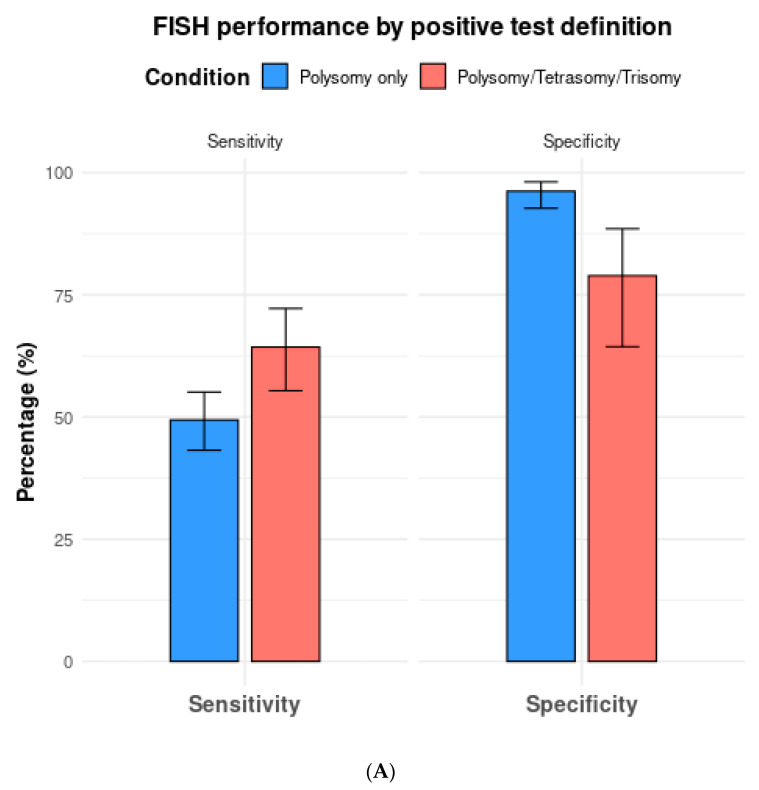

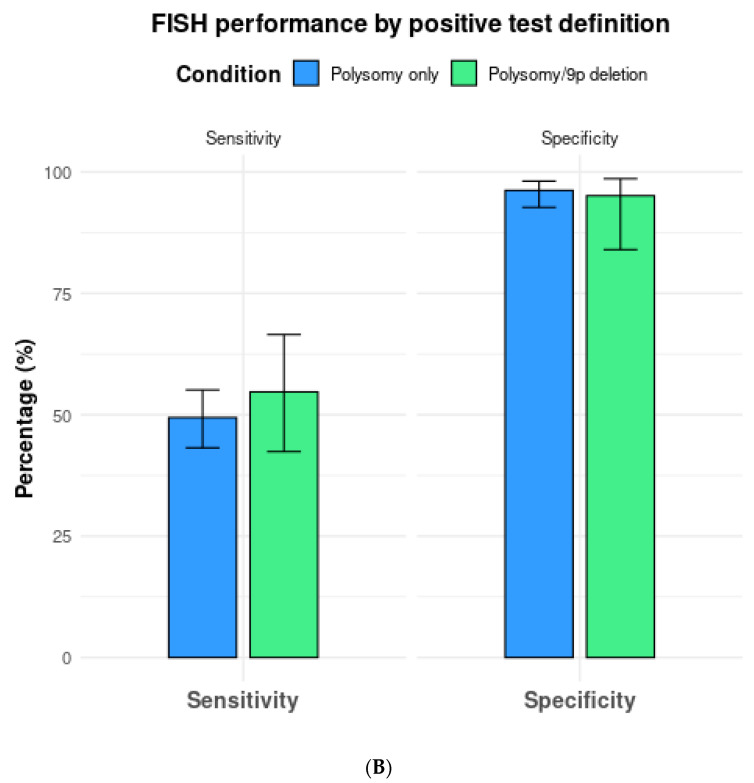
(**A**) Comparison of fluorescence in situ hybridization (FISH) performance based on positive test definitions of polysomy only vs. polysomy/tetrasomy/trisomy. (**B**) Comparison of fluorescence in situ hybridization (FISH) performance based on the positive test definitions of polysomy only vs. polysomy/9p deletion.

**Figure 5:**
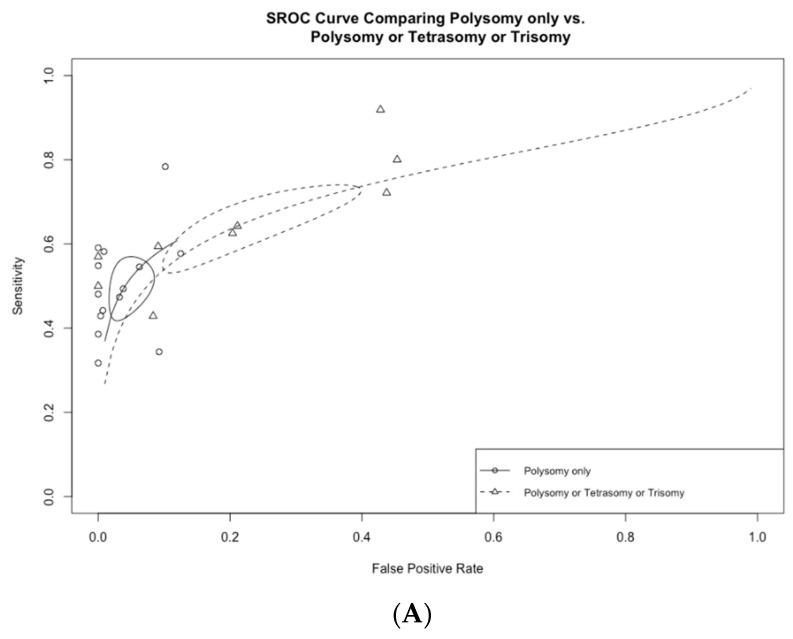

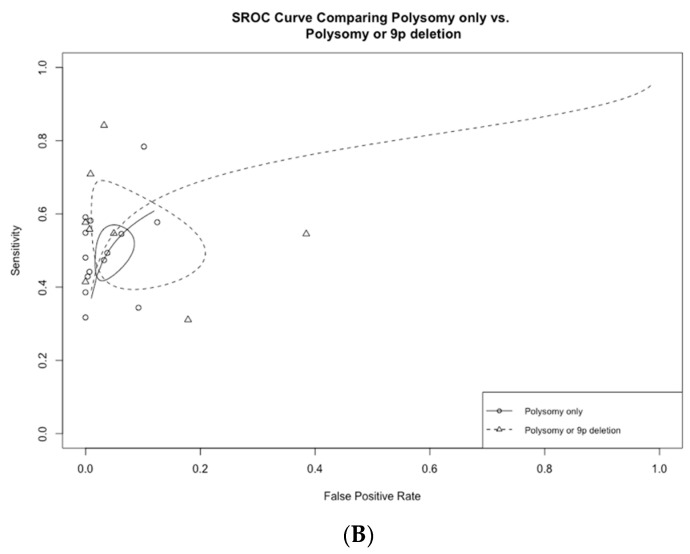
(**A**) Receiver operating curves comparing polysomy only vs. polysomy/tetrasomy/trisomy and (**B**) polysomy only vs. polysomy/9p deletion.

Among the 13 studies utilizing FISH polysomy as the threshold for diagnostic FISH, the overall test sensitivity was 49.4% (95% CI 43.2–55.5%), with a specificity of 96.2% (95% CI 92.7–98.1%) (Figure 4). The overall AUC was 0.74 (95% CI 0.72–0.76) (Figure 5A). On the other hand, in studies that considered polysomy, tetrasomy, or trisomy as positive FISH (n = 8), the overall sensitivity increased significantly (64.3%, 95% CI 55.4–72.2%) (Figure 4A). However, this increase in sensitivity was accompanied by a significant drop in specificity to 78.9% (95% CI 64.4–88.5%), with an overall AUC of 0.74 (95% CI 0.71–0.77).

In a total of seven studies that included 9p deletions in addition to polysomy as the criteria for a positive test, overall sensitivity, specificity, and AUC were 54.7% (95% CI 42.4–66.5%), 95.1% (95% CI 84.0–98.6%), and 0.77 (0.73–0.81), respectively (Table 2, Figure 4B).

When only including studies that compared two or more cutoffs, the sensitivity and specificity of FISH with polysomy only as the diagnostic threshold (n = 5 studies) were 52.9% (95% CI 44.2–61.4%) and 96.3% (95% CI 87.6–99.0%), respectively. The respective sensitivity and specificity for polysomy, tetrasomy, or trisomy were 65.7% (95% CI 57.3–73.1%) and 77.1% (95% CI 59.0–88.8%) (Table 2).

Meanwhile, the sensitivity and specificity of FISH in polysomy only vs. polysomy/9p deletion (n = 5 studies) were 46.3% (95% CI 38.6–54.2%) and 98.3% (95% CI 96.0–99.3%) vs. 61.0% (95% CI 47.7–72.9%) and 98.4% (95% CI 96.0–99.4%).

For studies including only patients with PSC (n = 3) [5,18,22], the overall sensitivity, specificity, and AUC of a positive FISH in detecting malignancy for biliary stricture were 64.3% (95% CI 19.5–93.0%), 88.2% (95% CI 52.6–98.0%), and 0.85 (95% CI 0.77–0.93), respectively.

## 4. Discussion

Molecular testing by UroVysion^®^ FISH has significantly improved the diagnosis of malignant biliary strictures and is currently recommended as part of multimodality sampling [4,33]. Although recommended, the definition of a diagnostic FISH test has not been standardized. Several abnormalities can be noted during the molecular FISH analysis (e.g., polysomy, tetrasomy, trisomy), thus creating a clinical dilemma that can dramatically affect a patient’s diagnosis and resulting management. In this systematic review and meta-analysis, the performance of FISH was found to vary significantly based on the definition of a positive test. A stricter threshold for diagnostic FISH tests, such as only considering polysomy, significantly increased specificity while maintaining sensitivity. Conversely, including tetrasomy or trisomy into the positive threshold significantly increased sensitivity but decreased specificity. Interestingly, adding 9p to the threshold improved sensitivity while maintaining specificity, compared to polysomy alone. Based on these findings, polysomy only or 9p deletion/polysomy should be considered as the criterion for defining a positive FISH test to improve diagnostic sensitivity while maintaining high specificity. It is important for molecular diagnostic testing for cancer to have a low rate of false-positive results (i.e., high specificity), which have significant clinical consequences. Thus, tetrasomy or trisomy should not be routinely regarded as a positive FISH test for malignancy based on the low specificity noted with this study.

The results of this meta-analysis reinforce the finding that FISH testing can increase the sensitivity of detecting malignant biliary strictures without reducing specificity [11]. Furthermore, FISH is known to be better at determining lesions deemed “suspicious for malignancy”. Despite the improved diagnostic performance, FISH testing should not be used as a standalone test; however, it is an excellent confirmatory test in addition to brush cytology for malignancy in patients with biliary stricture [24]. FISH results should be interpreted in the context of the individual patient and relevant clinical, laboratory (elevated CA19-9), and radiological features (e.g., dominant- or malignant-appearing stricture or an associated mass).

FISH polysomy and trisomy (most frequently CEP 7 probe) are the two most common FISH abnormalities within the biliary tract specimens. Cells demonstrating polysomy are chromosomally unstable by definition and hence are highly associated with malignancy. A polysomy FISH result is very specific for malignancy, with a PPV reaching 100% [12]. On the other hand, trisomy, particularly CEP 7, is observed in both neoplastic and non-neoplastic cells. The epidermal growth factor receptor gene is on chromosome 7 and is postulated to be involved in carcinogenesis. However, trisomy 7 has been observed in non-neoplastic inflammatory cells as well [34]. Thus, inclusion of trisomy into the definition of a positive test resulted in an increased sensitivity, with a significant drop in specificity. However, it must be noted that within the biliary tract, a subset, but not all, of patients with trisomy 7 will develop polysomy and, ultimately, CCA. Therefore, although not diagnostic, these patients should be closely monitored, and sampling should be repeated, as trisomy may reflect a pre-neoplastic state or dysplasia [34]. It must be noted that serial or multifocal polysomy is strongly associated with malignancy and should be factored into decision making in the context of the individual patient.

In this study, homozygous 9p deletion by FISH had a high specificity for malignancy, which was comparable to polysomy. In the FISH assay, the 9p21 probe spanned the *P16* tumor suppressor gene, frequently inactivated in human cancers, including CCA [35]. Although rare (in one study, 4% of CCAs had homozygous 9p deletion), this finding should raise concern for malignancy, even in the absence of polysomy, as the homozygous loss of 9p21 would be expected to result in an absence of p16 expression.

Compared to a previous meta-analysis assessing the diagnostic accuracy of FISH that demonstrated a sensitivity of 68% and specificity of 70% for the diagnosis of cholangiocarcinoma in PSC patients [36], this study reported a lower sensitivity but higher specificity for the diagnosis of malignancy in PSC patients. Overall, the performance characteristics of FISH in PSC individuals was similar to the overall population. Of note, most of the studies included in the previous meta-analysis were reported from the same institution and had overlapping/similar time periods, thus presenting a serious risk of bias, such as double counting samples.

In addition to FISH, several molecular techniques have been developed over the last decade to personalize the diagnosis of malignancy in biliary strictures. NGS for the mutational analysis of both biliary brushings and bile has been demonstrated to be a sensitive diagnostic tool in both patients with and without PSC [37]. A 28-gene NGS panel demonstrated a diagnostic sensitivity of 75% for malignant strictures on both biliary brushings and biliary biopsies [38]. An additional advantage of NGS is the ability to identify actionable mutations that may guide therapy in these aggressive malignancies. Furthermore, the NGS of cell-free DNA in bile collected at the time of ERCP resulted in a sensitivity of 60% for the diagnosis of malignancy [39]. It must be noted that mutations may be seen in specimens from benign strictures, which creates a clinical dilemma regarding management, especially in the background of inflammation-induced cellular damage.

Aberrant DNA methylation, a key epigenetic event in carcinogenesis, has also been studied to aid in the diagnosis of malignant biliary strictures [40]. Digital droplet polymerase chain reaction (ddPCR)-based analysis of the methylation index of targeted genes has shown high sensitivity and specificity for malignancy in both brushings and bile specimens [41,42]. DNA methylation is purported to be less resource intensive and easier to perform than NGS and FISH. However, the clinical utility of these targeted assays can only be ascertained after external validations, which are ongoing [43]. Additionally, studies are needed to compare different molecular techniques and create appropriate decision trees for choosing the right test for the right clinical scenario.

Concurrently, advancements in per oral cholangioscopy for the direct visualization of the biliary tract has improved the classification of biliary strictures. Visual findings such as abnormal mucosal features, papillary projections, and abnormal vessels have been reported to have high sensitivity for the diagnosis of malignancy, with good interobserver agreement when using standard classification systems [44]. However per oral cholangioscopy has limited availability due to high cost and the technical expertise needed, with a trend towards higher adverse events in comparison with traditional ERCP-based sampling [45].

The strengths of this study include a rigorous selection of studies to avoid the risk of double counting. Furthermore, the study employed the most up-to-date DTA meta-analysis statistics endorsed by the Cochrane Collaboration [46]. However, there are several limitations to this study. All the FISH studies included in this meta-analysis used the UroVysion^®^ probe set. A newer PB FISH test is purported to have an augmented diagnostic value, compared to the UroVysion^®^ probe set, but there are limited published studies to perform a rigorous meta-analysis [33,47]. Studies using the PB FISH probe set were excluded, as these tests are not widely available and have inherent differences in performance characteristics; therefore, their inclusion would introduce significant bias in the meta-analysis. The notable heterogeneity observed based on prediction ellipses from SROC curves (Figure 3B) were likely a result of differences in the patient population, prevalence of malignancy, and thresholds used in each study. Additionally, a comparison of FISH performance with brush cytology or newer techniques, such as NGS, was not available, as the studies lacked paired data. Additionally, due to the lack of follow-up data, whether individuals with abnormalities other than polysomy went on to develop malignancy could not be ascertained and should be studied in future studies.

In conclusion, a positive FISH definition of polysomy or 9p deletion with polysomy has the best performance characteristics, with moderate sensitivity and high specificity for detecting malignancy among patients with biliary strictures. Given the low diagnostic specificity, FISH tetrasomy or trisomy should not be routinely considered to be a positive FISH result.

## Figures and Tables

**Table 1:** Baseline characteristics of the included studies.

Study	Location	Study Period	Study Threshold for Diagnostic FISH	FISH Samples (n)	Malignancy (n)	Cholangiocarcinoma (n)
Kipp 2004 [5]	USA	June 2000–June 2002	Polysomy only	129	64	39
Barr Fritcher 2009 [17]	USA	October 2003–March 2006	Polysomy, Tetrasomy, or Trisomy Polysomy only	484	219	152
Gonda 2012 [27]	USA	February 2008–February 2010	Polysomy or 9p deletion Polysomy only	50	19	16
Smoczynski 2012 [28]	Poland	September 2008–August 2010	Polysomy or Trisomy	47	35	11
Boldorini 2015 [29]	Italy	June 2007–September 2009	Polysomy or 9p deletion Polysomy only	71	52	NA
Eaton 2015 [18]	USA	January 2005–July 2013	Polysomy, Tetrasomy, or Trisomy Polysomy only	371	37	37
Nanda 2015 [19]	USA	December 2008–November 2012	Polysomy only	50	22	22
Chaiteerakij 2016 [30]	Thailand	March 2010–December 2013	Polysomy or Trisomy with 9p deletion Polysomy only	99	93	58
Dudley 2016 [20]	USA	April 2014–January 2015	Polysomy only	81	33	10
Gonda 2017 [21]	USA	June 2012–June 2014	Polysomy or 9p deletion Polysomy only	100	41	13
Wu 2017 [31]	China	October 2008–June 2009	Polysomy or Trisomy	27	22	17
Brooks 2018 [22]	USA	2006–2016	Polysomy or Trisomy or 9p deletion Polysomy or 9p deletion Polysomy only	281	138	55
Zhai 2018 [23]	USA	Not Reported	Polysomy or 9p deletion	35	22	3
Kushnir 2019 [24]	USA	November 2013–February 2016	Polysomy only	101	70	33
Han 2021 [6]	USA	January 2008–July 2015	Polysomy, Tetrasomy, or 9p deletion	126	40	NA
MettMan 2021 [25]	USA	October 2014–November 2019	Polysomy or 9p deletion Polysomy only	169	55	NA
Khan 2022 [26]	USA	January 2001–September 2019	Polysomy or Trisomy Polysomy only	193	97	NA
Zoundjiekpon 2023 [32]	Czech Republic	April 2019–January 2021	Polysomy or 9p deletion	102	74	26

**Table 2:** Diagnostic performance of fluorescence in situ hybridization (FISH) based on the definition of a positive test.

	Sensitivity	Specificity	Positive LR	Negative LR	DOR
All included studies					
All thresholds (n = 18)	57.6 (49.4–65.4)	87.8 (79.2–93.2)	4.9 (2.9–8.1)	0.49 (0.40–0.57)	10.3 (5.4–17.9)
Polysomy only (n = 13)	49.4 (43.2–55.1)	96.2 (92.7–98.1)	13.7 (7.0–24.5)	0.53 (0.47–0.59)	26.1 (12.9–47.2)
Polysomy, tetrasomy, or trisomy (n = 8)	64.3 (55.4–72.2)	78.9 (64.4–88.5)	2.8 (1.8–4.4)	0.47 (0.41–0.53)	6.0 (3.5–9.7)
Polysomy or 9p deletion (n = 7)	54.7 (42.4–66.5)	95.1 (84.0–98.6)	14.0 (3.1–42.6)	0.48 (0.35–0.65)	31.7 (5.0–109.0)
Studies with head-to-head comparison of different thresholds					
Polysomy only (n = 5)	52.9 (44.2–61.4)	96.3 (87.6–99.0)	17.1 (4.8–45.7)	0.49 (0.42–0.57)	34.2 (10.1–86.1)
Polysomy, tetrasomy, or trisomy (n = 5)	65.7 (57.3–73.1)	77.1 (59.0–88.8)	3.0 (1.8–5.3)	0.45 (0.39–0.52)	6.8 (3.6–11.7)
Polysomy only (n = 5)	46.3 (38.6–54.2)	98.3 (96.0–99.3)	29.2 (11.6–61.8)	0.55 (0.47–0.63)	54.1 (20.1–118.0)
Polysomy or 9p deletion (n = 5)	61.0 (47.7–72.9)	98.4 (96.0–99.4)	43.0 (16.0–94.8)	0.40 (0.28–0.53)	110.0 (40.2–242.0)

Notes: DOR: diagnostic odds ratio; LR: likelihood ratio. Data presented as % or n (95% CI).

## Data Availability

The data are available upon reasonable request from the first or corresponding author.

## Supplementary Materials

The following supporting information can be downloaded at: https://www.mdpi.com/article/10.3390/jcm13216457/s1, Supplementary Materials S1. PRISMA-DTA Checklist [48]. Supplementary Materials S2. Search Strategy. Supplementary Materials S3. Risk of Bias Assessment with the QUADAS-2 Tool. Supplementary Materials S4. Diagnostic Accuracy of FISH for detection of Malignancy. Supplementary Materials S5. Funnel plot with Deek’s Linear Test for Publication Bias (*p* = 0.572). Supplementary Materials S6. Summary receiver operating curves for only patients with PSC.

## Abbreviations

CI	Confidence Interval
DOR	Diagnostic Odds Ratio
DTA	Diagnostic Test Accuracy
FISH	Fluorescence In Situ Hybridization
LR	Likelihood Ratio
PB	Pancreaticobiliary
PSC	Primary Sclerosing Cholangitis
ROC	Receiver Operating Characteristic Curve
QUADAS-2	Quality Assessment of Diagnostic Accuracy Studies-2
SROC	Summary Receiver Operating Characteristic Curve
