# Supplementary material for: Diagnostic Accuracy Performance of Fluorescence In Situ Hybridization (FISH) for Biliary Strictures: A Systematic Review and Meta-Analysis
Source: J Clin Med. 2024 Oct 28;13(21):6457. doi: 10.3390/jcm13216457 (PMC11546496; doi:10.3390/jcm13216457)

Supplementary Materials

Supplementary Materials S1. PRISMA-DTA Checklist

Supplementary Materials S2. Search Strategy

Supplementary Materials S3. Risk of Bias Assessment with the QUADAS-2 Tool

Supplementary Materials S4. Diagnostic Accuracy of FISH for detection of Malignancy

Supplementary Materials S5. Funnel plot with Deek's Linear Test for Publication Bias (p=0.572)

Supplementary Materials S6. Summary receiver operating curves for only patients with PSC

PRISMA 2020 Main Checklist

Topic	No.	Item	Location where item is reported
**TITLE**			
**Title**	1	Identify the report as a systematic review.	Section 1, Page 1
**ABSTRACT**			
**Abstract**	2	See the PRISMA 2020 for Abstracts checklist	
**INTRODUCTION**			
**Rationale**	3	Describe the rationale for the review in the context of existing knowledge.	Introduction, Line 14-16
**Objectives**	4	Provide an explicit statement of the objective(s) or question(s) the review addresses.	Introduction line 18-20
**METHODS**			
**Eligibility criteria**	5	Specify the inclusion and exclusion criteria for the review and how studies were grouped for the syntheses.	Page 6, Line 10-16
**Information sources**	6	Specify all databases, registers, websites, organisations, reference lists and other sources searched or consulted to identify studies. Specify the date when each source was last searched or consulted.	Page 6, Line 7-8
**Search strategy**	7	Present the full search strategies for all databases, registers and websites, including any filters and limits used.	Appendix-1
**Selection process**	8	Specify the methods used to decide whether a study met the inclusion criteria of the review, including how many reviewers screened each record and each report retrieved, whether they worked independently, and if applicable, details of automation tools used in the process.	page 7, Line 15-24
**Data collection process**	9	Specify the methods used to collect data from reports, including how many reviewers collected data from each report, whether they worked independently, any processes for obtaining or confirming data from study investigators, and if applicable, details of automation tools used in the process.	Page 7, Line 17-20

Topic	No.	Item	Location where item is reported
**Data items**	10a	List and define all outcomes for which data were sought. Specify whether all results that were compatible with each outcome domain in each study were sought (e.g. for all measures, time points, analyses), and if not, the methods used to decide which results to collect.	Page 7, Line 8-13
	10b	List and define all other variables for which data were sought (e.g. participant and intervention characteristics, funding sources). Describe any assumptions made about any missing or unclear information.	Page 7, Line 14-19
**Study risk of bias assessment**	11	Specify the methods used to assess risk of bias in the included studies, including details of the tool(s) used, how many reviewers assessed each study and whether they worked independently, and if applicable, details of automation tools used in the process.	Page 7, Line 20-21
**Effect measures**	12	Specify for each outcome the effect measure(s) (e.g. risk ratio, mean difference) used in the synthesis or presentation of results.	Page 8, Line 4-7
**Synthesis methods**	13a	Describe the processes used to decide which studies were eligible for each synthesis (e.g. tabulating the study intervention characteristics and comparing against the planned groups for each synthesis (item 5)).	Table 1, Figure 1
	13b	Describe any methods required to prepare the data for presentation or synthesis, such as handling of missing summary statistics, or data conversions.	NA
	13c	Describe any methods used to tabulate or visually display results of individual studies and syntheses.	Table 1, Figure 1-4
	13d	Describe any methods used to synthesize results and provide a rationale for the choice(s). If meta-analysis was performed, describe the model(s), method(s) to identify the presence and extent of statistical heterogeneity, and software package(s) used.	Page 8, Line 2-17
	13e	Describe any methods used to explore possible causes of heterogeneity among study results (e.g. subgroup analysis, meta-regression).	Page 8, Line 14-16
	13f	Describe any sensitivity analyses conducted to assess robustness of the synthesized results.	Page 8
**Reporting bias assessment**	14	Describe any methods used to assess risk of bias due to missing results in a synthesis (arising from reporting biases).	NA

Topic	No.	Item	Location where item is reported
**Certainty assessment**	15	Describe any methods used to assess certainty (or confidence) in the body of evidence for an outcome.	NA
**RESULTS**			
**Study selection**	16a	Describe the results of the search and selection process, from the number of records identified in the search to the number of studies included in the review, ideally using a flow diagram.	Figure 1
	16b	Cite studies that might appear to meet the inclusion criteria, but which were excluded, and explain why they were excluded.	Page 6, Line 12-15
**Study characteristics**	17	Cite each included study and present its characteristics.	Table-1
**Risk of bias in studies**	18	Present assessments of risk of bias for each included study.	Figure 2
**Results of individual studies**	19	For all outcomes, present, for each study: (a) summary statistics for each group (where appropriate) and (b) an effect estimate and its precision (e.g. confidence/credible interval), ideally using structured tables or plots.	Table-1
**Results of syntheses**	20a	For each synthesis, briefly summarise the characteristics and risk of bias among contributing studies.	Fugure 3
	20b	Present results of all statistical syntheses conducted. If meta-analysis was done, present for each the summary estimate and its precision (e.g. confidence/credible interval) and measures of statistical heterogeneity. If comparing groups, describe the direction of the effect.	Page 9-10
	20c	Present results of all investigations of possible causes of heterogeneity among study results.	NA
	20d	Present results of all sensitivity analyses conducted to assess the robustness of the synthesized results.	Page 10-11
**Reporting biases**	21	Present assessments of risk of bias due to missing results (arising from reporting biases) for each synthesis assessed.	Line XX-ZZ
**Certainty of evidence**	22	Present assessments of certainty (or confidence) in the body of evidence for each outcome assessed.	Line XX-ZZ
**DISCUSSION**			

Topic	No.	Item	Location where item is reported
**Discussion**	23a	Provide a general interpretation of the results in the context of other evidence.	Page 12, Line 2-20
	23b	Discuss any limitations of the evidence included in the review.	Page 14, line 16-23
	23c	Discuss any limitations of the review processes used.	Page 14, Line 16-18
	23d	Discuss implications of the results for practice, policy, and future research.	Page 15, line 4-8
**OTHER INFORMATION**			
**Registration and protocol**	24a	Provide registration information for the review, including register name and registration number, or state that the review was not registered.	Methods
	24b	Indicate where the review protocol can be accessed, or state that a protocol was not prepared.	Register
	24c	Describe and explain any amendments to information provided at registration or in the protocol.	NA
**Support**	25	Describe sources of financial or non-financial support for the review, and the role of the funders or sponsors in the review.	Title page
**Competing interests**	26	Declare any competing interests of review authors.	Title page
**Availability of data, code and other materials**	27	Report which of the following are publicly available and where they can be found: template data collection forms; data extracted from included studies; data used for all analyses; analytic code; any other materials used in the review.	Title page

PRIMSA Abstract Checklist

Topic	No.	Item	Reported?
**TITLE**			
**Title**	1	Identify the report as a systematic review.	Yes
**BACKGROUND**			

Topic	No.	Item	Reported?
**Objectives**	2	Provide an explicit statement of the main objective(s) or question(s) the review addresses.	Yes
**METHODS**			
**Eligibility criteria**	3	Specify the inclusion and exclusion criteria for the review.	Yes
**Information sources**	4	Specify the information sources (e.g. databases, registers) used to identify studies and the date when each was last searched.	Yes
**Risk of bias**	5	Specify the methods used to assess risk of bias in the included studies.	Yes
**Synthesis of results**	6	Specify the methods used to present and synthesize results.	Yes
**RESULTS**			
**Included studies**	7	Give the total number of included studies and participants and summarise relevant characteristics of studies.	Yes
**Synthesis of results**	8	Present results for main outcomes, preferably indicating the number of included studies and participants for each. If meta-analysis was done, report the summary estimate and confidence/credible interval. If comparing groups, indicate the direction of the effect (i.e. which group is favoured).	Yes
**DISCUSSION**			
**Limitations of evidence**	9	Provide a brief summary of the limitations of the evidence included in the review (e.g. study risk of bias, inconsistency and imprecision).	Yes
**Interpretation**	10	Provide a general interpretation of the results and important implications.	Yes
**OTHER**			
**Funding**	11	Specify the primary source of funding for the review.	Yes
**Registration**	12	Provide the register name and registration number.	Yes

From: Page MJ, McKenzie JE, Bossuyt PM, Boutron I, Hoffmann TC, Mulrow CD, et al. The PRISMA 2020 statement: an updated guideline for reporting systematic reviews. MetaArXiv. 2020, September 14. DOI: 10.31222/osf.io/v7gm2. For more information, visit: www.prisma-statement.org

Supplementary Materials S2. Search Strategy

Ovid

Database(s): EBM Reviews - Cochrane Central Register of Controlled Trials July 2024, EBM Reviews - Cochrane Database of Systematic Reviews 2005 to August 28, 2024, Embase 1974 to 2024 August 30, Ovid MEDLINE(R) and Epub Ahead of Print, In-Process, In-Data-Review & Other Non-Indexed Citations, Daily and Versions 1946 to August 29, 2024
Search Strategy:

#	Searches	Results
1	exp Biliary Tract/	782979
2	exp Biliary Tract Diseases/	410358
3	biliary.ti,ab,kf.	249231
4	1 or 2 or 3	1163925
5	(constrict* or obstruct* or stenos* or stricture*).ti,ab,kf.	1519290
6	4 and 5	87441
7	(brush or brushes or brushing or brushings).ti,ab,kf.	87027
8	6 and 7	1791
9	cytology.fs.	1048468
10	exp Cytological Techniques/	2654380
11	exp Cholangiopancreatography, Endoscopic Retrograde/	72329
12	(cholangiopancreatograph* or Cytological or cytology or "endoscopic cholangiograph*" or "endoscopic cholangiopancreatograph*" or "endoscopic pancreatocholangiograph*" or "endoscopic pancreatograph*" or "endoscopic retrograde cholangiograph*" or "endoscopic retrograde cholangiopancreatograph*" or "endoscopic retrograde pancreaticocholangiograph*" or "endoscopic retrograde pancreatocholangiograph*" or ERCP or pancreaticocholangiograph* or pancreatocholangiograph* or "retrograde cholangiopancreatograph*" or "retrograde endoscopic cholangiograph*" or "retrograde endoscopic cholangiopancreatograph*").ti,ab,kf.	282012
13	9 or 10 or 11 or 12	3613492
14	8 and 13	1677
15	exp Neoplasms/	9977124
16	((("bile duct" or "bile ducts" or "bile tract*" or biliary or "gall duct" or "gall ducts" or cholangiocellular or cholangiolar) adj3 (carcinoma* or tumor* or tumour*)) or (positive adj3 (result* or yield* or test* or diagnos*)) or benign or cancer* or CCA or cholangiocarcinoma* or "Klatskin tumor*" or "Klatskin tumour*" or malignan* or neoplas*).ti,ab,kf.	8491423
17	15 or 16	12522366
18	14 and 17	1599

19	limit 18 to (conference abstract or editorial or erratum or note or addresses or autobiography or bibliography or biography or blogs or comment or dictionary or directory or interactive tutorial or interview or lectures or legal cases or legislation or news or newspaper article or overall or patient education handout or periodical index or portraits or published erratum or webcasts) [Limit not valid in CCTR,CDSR,Embase,Ovid MEDLINE(R); records were retained]	718
20	18 not 19	881
21	remove duplicates from 20	532

Scopus

1 TITLE-ABS-KEY(biliary AND (constrict* OR obstruct* OR stenos* OR stricture*))
2 TITLE-ABS-KEY(brush OR brushes OR brushing OR brushings)
3 TITLE-ABS-KEY(cholangiopancreatograph* OR Cytological OR cytology OR "endoscopic cholangiograph*" OR "endoscopic cholangiopancreatograph*" OR "endoscopic pancreatocholangiograph*" OR "endoscopic pancreatograph*" OR "endoscopic retrograde cholangiograph*" OR "endoscopic retrograde cholangiopancreatograph*" OR "endoscopic retrograde pancreaticocholangiograph*" OR "endoscopic retrograde pancreatocholangiograph*" OR ERCP OR pancreaticocholangiograph* OR pancreatocholangiograph* OR "retrograde cholangiopancreatograph*" OR "retrograde endoscopic cholangiograph*" OR "retrograde endoscopic cholangiopancreatograph*")
4 TITLE-ABS-KEY((("bile duct" or "bile ducts" or "bile tract*" or biliary or "gall duct" or "gall ducts" or cholangiocellular or cholangiolar) W/3 (carcinoma* or tumor* or tumour*)) OR (positive W/3 (result* or yield* or test* or diagnos*)) OR benign OR cancer* OR CCA OR cholangiocarcinoma* OR "Klatskin tumor*" OR "Klatskin tumour*" OR malignan* OR neoplas*)
5 1 and 2 and 3 and 4
6 DOCTYPE(ab) OR DOCTYPE(ed) OR DOCTYPE(bk) OR DOCTYPE(er) OR DOCTYPE(no) OR DOCTYPE(sh)
7 5 and not 6
8 INDEX(embase) OR INDEX(medline) OR PMID(0* OR 1* OR 2* OR 3* OR 4* OR 5* OR 6* OR 7* OR 8* OR 9*)
9 7 and not 8

Supplementary Materials S3. Risk of Bias Assessment with the QUADAS-2 Tool

Study	Risk of Bias				Applicability Concerns		
	Patient Selection	Index Test	Reference Standard	Flow and Timing	Patient Selection	Index Test	Reference Standard
**Kipp 2004**	Low	Low	Low	Low	Low	Low	Low
**Barr Fritcher 2009**	Low	Low	Low	Low	Low	Low	Low
**Gonda 2012**	Low	Low	Low	Low	Low	Low	Low
**Smoczynski 2012**	Low	Low	Low	Low	Low	Low	Low
**Boldorini 2015**	Low	Low	Low	Low	Low	Low	Low
**Eaton 2015**	Low	Low	Low	Low	Low	Low	Low
**Nanda 2015**	Low	Low	Low	Low	Low	Low	Low
**Chaiteerakij 2016**	Low	Low	Low	Low	Low	Low	Low
**Dudley 2016**	Low	Low	Low	Low	Low	Low	Low
**Gonda 2017**	Low	Low	Low	Low	Low	Low	Low
**Wu 2017**	Low	Low	Low	Low	Low	Low	Low
**Brooks 2018**	Low	Low	Low	Low	Low	Low	Low
**Zhai 2018**	Low	Low	Low	Low	Low	Low	Low
**Kushnir 2019**	Low	Low	Low	Low	Low	Low	Low
**Han 2021**	Low	Low	Low	Low	Low	Low	Low
**MettMan 2021**	Low	Low	Low	Low	Low	Low	Low
**Khan 2022**	Low	Low	Low	Low	Low	Low	Low
**Zoundjiekpon 2023**	Low	Low	Low	Low	Low	Low	Low

Supplementary Materials S4. Diagnostic Accuracy of FISH for Detection of Malignancy

Study	TP	FP	FN	TN	Total	Prevalence of Malignancy
**Kipp 2004**	22	6	42	59	129	0.50
**Barr Fritcher 2009**	137	54	82	211	484	0.45
**Gonda 2012**	16	1	3	30	50	0.38
**Smoczynski 2012**	15	1	20	11	47	0.74
**Boldorini 2015**	30	0	22	19	71	0.73
**Eaton 2015**	34	143	3	191	371	0.10
**Nanda 2015**	13	0	9	28	50	0.44
**Chaiteerakij 2016**	53	0	40	6	99	0.94
**Dudley 2016**	18	3	15	45	81	0.41
**Gonda 2017**	17	0	24	59	100	0.41
**Wu 2017**	11	0	11	5	27	0.81
**Brooks 2018**	82	13	56	130	281	0.49
**Zhai 2018**	12	5	10	8	35	0.63
**Kushnir 2019**	27	0	43	31	101	0.69
**Han 2021**	32	39	8	47	126	0.32
**MettMan 2021**	39	1	16	113	169	0.33
**Khan 2022**	70	42	27	54	193	0.50
**Zoundjiekpon 2023**	23	5	51	23	102	0.73

Notes

FN: False Negative; FP: False Positive; TN: True Negative; TP: True Positive

Supplementary Materials S5. Funnel plot with Deek's Linear Test for Publication Bias (p=0.344)

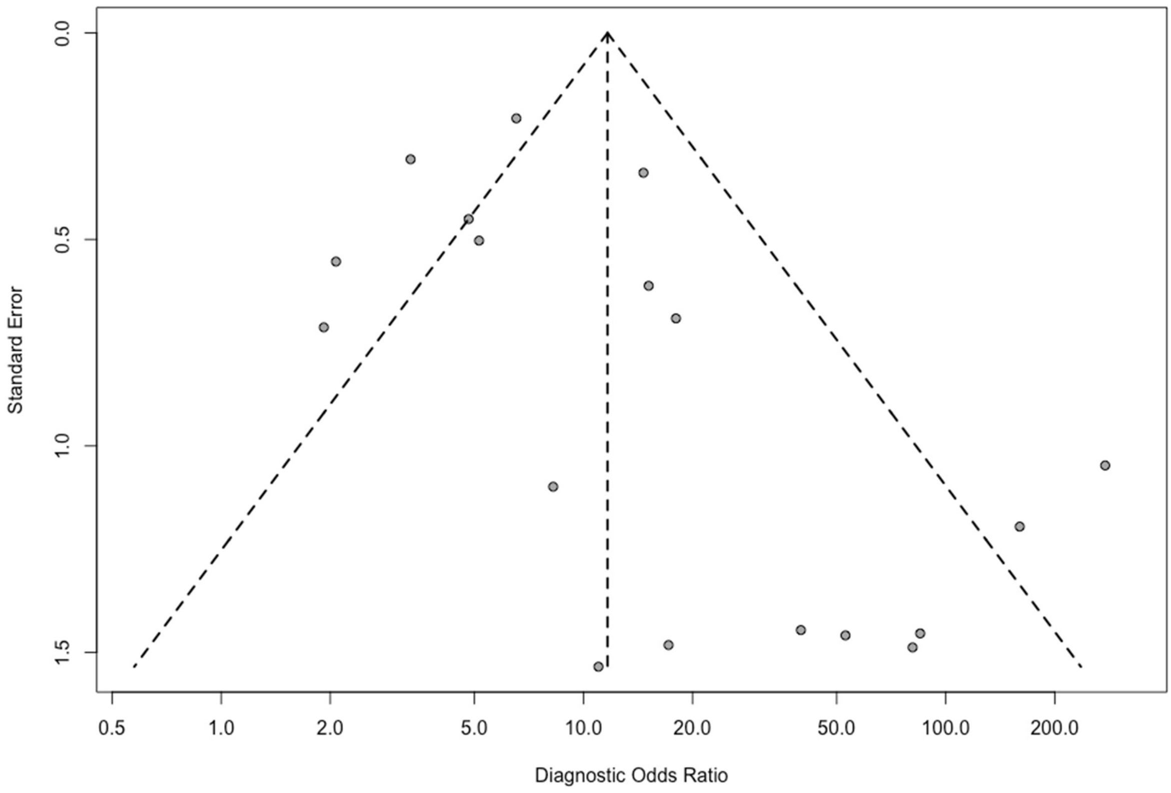

Supplementary Materials S6. Summary receiver operating curves for only patients with PSC

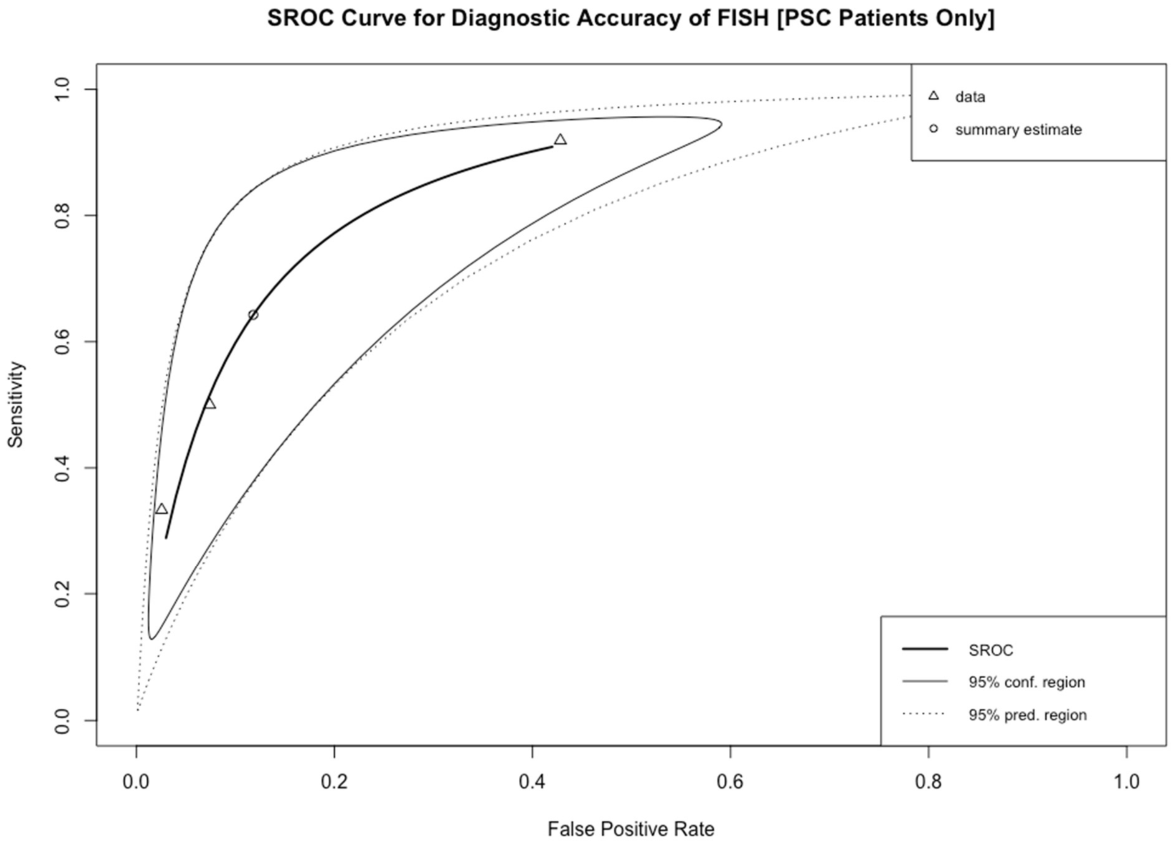

Supplement: Supplementary file 1 [file jcm-13-06457-s001.zip › jcm-3266394-supplementary.pdf]
